# Profiling of genes associated with the murine model of oxygen-induced retinopathy

**Published:** 2013-04-03

**Authors:** Xia Yang, Xiaoguang Dong, Changkai Jia, Yiqiang Wang

**Affiliations:** 1 Qingdao University-SEI Joint Ophthalmology Program, Shandong Eye Institute, Shandong Academy of Medical Sciences, Qingdao, China; 2 Shandong Provincial Key Laboratory of Ophthalmology, Shandong Eye Institute, Shandong Academy of Medical Sciences, Qingdao, China

## Abstract

**Purpose:**

To compare the clinical features and gene expression patterns of the physiologic development of retinal vessels and oxygen-induced retinopathy (OIR) in a mouse model, with the aim of identifying differential regulators of physiologic and pathological angiogenesis in the retina.

**Methods:**

C57BL/6J mice were used. Seven-day-old pups were subjected to OIR induction following the standard protocols of entering a hyperoxic chamber on day 7 (P7) and returning to a normoxic condition (relative hypoxia) on day 12 (P12). The retinal vasculatures in the OIR model 24 h (P8-O) or 5 days (P12-O) after switching to the hyperoxic environment and 24 h (P13-O) after returning to normoxic conditions were evaluated with retinal flat mounts and compared with those of age-matched controls (i.e., P8-N, P12-N, P13-N). Gene expression profiling was performed using Phalanx Mouse Whole Genome OneArray microarrays. Normal 9-day-old mice were considered representative of physiologic angiogenesis and compared with 30-day-old mice. A bioinformatics analysis was performed on differentially expressed genes using various comparisons, and real-time reverse-transcription PCR was used to confirm the changes in the genes of interest.

**Results:**

The sequential orders and patterns of vasculature development in normal mice and the OIR models were significantly different. In brief, in the early days (P1 to P7) for normal mice, retinal vessels grew from the optic disc into the non-vascularized retina in a radial fashion. In the hyperoxic stage of the OIR model, the main central retina became devoid of a vascular network, and when the mice returned to the normoxic room, the vessels grew from peripheral perfused areas toward the center of the retina, but the development of intermediate and deep layers of vasculature was significantly delayed. Gene profiling at three critical time points (P8, P12, and P13) showed that 162 probes were upregulated to ≥1.5-fold or downregulated to ≤0.67-fold at one or more time points in the OIR model compared to the controls. In the 45 upregulated genes for the P8-O/P8-N group, enriched genes were mainly related to cytoskeleton formation, whereas the 62 upregulated genes for P13-O/P13-N participated in various pathological processes. In the physiologic conditions on P9, however, 135 genes were upregulated compared with P30; the gap junction and Fc gamma R-mediated phagocytosis were the two main enriched pathways for these genes. Fifty-three probes, including vascular endothelium growth factor A, annexin A2, and endothelin 2, changed at P13-O but not at P9-N, and these changed genes might reflect the modulation of pathological neovascularization.

**Conclusions:**

Angiogenesis in physiologic and pathological conditions is characterized by the differential presentation of vasculature and gene expression patterns. Investigation of those genes unique to the OIR model may help develop new strategies and therapies for intervening in retinal neovascularization.

## Introduction

Retinal neovascularization (RNV) is a common pathological process that occurs in the later stages of several blinding retinal disorders, including retinopathy of prematurity, diabetic retinopathy, and retinal vein occlusion. RNV comprises a complex cascade of molecular and cellular events, among which the oxygen supply-related pathway plays an important role, regardless of the original etiological factors. Specifically, retinal ischemia induced by disturbances in vascular function acts as an angiogenesis mediator. Unlike the knowledge of choroidal neovascularization, which has come mainly from studies using human subjects and, to a lesser extent, laser-induced choroidal neovascularization in rodents, the main body of knowledge of RNV is from rodent models of oxygen-induced retinopathy (OIR). This model faithfully mimics two phases of retinopathy of prematurity—the initial vasoobliteration and subsequent neovascularization. Using this model, the role of many angiogenic factors such as vascular endothelial growth factor (*Vegf*) [1], angiopoietin-2 [2], hypoxia-inducible factor-1 (*Hif1*) [3], tumor necrosis factor [4], nuclear factor-κB [5], and insulin-like growth factor-1 [6] has been shown in RNV pathogenesis. Among these, *Vegf* has attracted the most attention, and strategies targeting this molecule have been successfully used in managing neovascularization in and out of ocular environments [7-9]. Other potential players in various RNVs remain to be defined. In the project described here, we started by comparing the vessels’ growth patterns in normal-developing retinas and in the murine OIR model, followed by profiling the gene expression patterns at various stages of these physiologic and pathological conditions. While this study was in progress, three similar studies, all using a microarray strategy and OIR models, were published [10-12]. Even though similar protocols were used for the OIR models, there were major differences in the results described below and those of the three previous reports, and the current report emphasizes the difference in pathological and physiologic angiogenesis at the histological and molecular levels.

## Methods

### Animal model

Wild-type C57BL/6J mice were used in this study. Normal controls were raised in normal air from birth. The OIR model that was used [13,14] involves transferring newborn pups at postnatal day 7 (P7) and their mothers to chambers supplied with 75±2% oxygen under continual monitoring with an LBCY-12C oximeter (Lubo WeiYe Environmental Protection Technology Co., Ltd, Qingdao, China). After 5 days, the animals returned to and remained in normal air until they were euthanized. At different time points, mice were anaesthetized using intraperitoneal injection of a mixture of 50 mg/kg ketamine (Gutian Pharmaceutical, Fujian, China) and 10 mg/kg chlorpromazine hydrochloride (Harvest Pharmaceutical, Shanghai, China). The retinas were harvested for individual assay as described below. The use of the animals was approved by the institution and adhered to the Association for Research in Vision and Ophthalmology Statement for the Use of Animals in Ophthalmic and Vision Research.

### Retinal flat mounts with fluorescein isothiocyanate-dextran perfusion

Anesthetized mice were perfused through the precava with fluorescein isothiocyanated dextran of a molecular weight of 2,000 kDa (Sigma Aldrich, St. Louis, MO). The eyes were enucleated and fixed in 4% paraformaldehyde for 10 to 30 min, depending on the age of the mice. Retinal flat mounts were prepared following routine protocols in this laboratory and examined with a C1-plus confocal fluorescence microscope (Nikon, Tokyo, Japan). Fine adjustment was carefully used to capture the signals produced by vessels in different levels of the retina, and as much of the area of the retina was observed as possible. At least three retinas were included in each group at each time point.

### Microarray analysis

At 24 h or 5 days after entering the hyperoxic chamber (P8 and P12, respectively) and 24 h after returning to a normoxic atmosphere (P13), the animals were euthanized via cervical dislocation under anesthesia, and their eyeballs removed. Ten neural retinas from five mice were harvested and pooled into one sample. Control samples of age-matched normal mice were similarly prepared. To evaluate the gene expression patterns in the physiologically developing retinal vasculature system, we also compared the gene expressions of normal retinas at day 9 (P9) and P30. These two time points were selected because the physiologic retinal vasculature develops quickly at P9, whereas at P30 the retinal vasculature is mature and stable. Due to the limited number of pups in most litters, the five animals in the same experimental sample were originally from the same litter, but the mice for different samples in all groups were from different litters. For all samples, total RNA was isolated using NucleoSpin RNA clean-up columns (Macherey-Nagel, Düren, Germany). All remaining processes after RNA extraction, up to the preliminary analysis of microarray data, were performed via custom service at Phalanx Biotech Group, Inc. (Hsinchu, Taiwan). Measurements taken using the Agilent 2100 Bioanalyzer (Agilent Technologies, Santa Clara, CA), and gel electrophoresis was used to ensure the quality of all RNA samples. The RNA integrity numbers were greater than 8.0 and the A_260_/A_280_ greater than 1.91 for all samples, indicating that they were high-quality samples suitable for microarray assay. The total RNA of all 24 samples averaged at 70.3±43.0 μg. A dual labeling and microarray strategy was exploited, with two samples for comparison labeled with Cy5 or Cy3 fluorescence, respectively, but hybridized onto the same microarray. For this purpose, 2.5 μg of RNA from paired samples were subjected to cRNA amplification and complementary RNA labeling with Cy5 or Cy3 fluorescence using an Ambion MessageAmp aRNA kit (Life Technologies, Carlsbad, CA). To eliminate potential bias caused by fluorescence labeling, a fluorescence switch was applied to all pairs. After spectrophotometric quantification (above), an equal amount of labeled sample pairs (10 μg each) was mixed and hybridized onto Phalanx Mouse Whole Genome OneArray Microarrays (Phalanx Biotech Group, Inc.). These arrays had 31,798 probes in total, including 1,876 internal controls and 29,922 for transcripts covering the whole genome, based on the Mouse Exonic Evidence Based Oligonucleotide [15]. Among the transcripts, 21,973 (73.4%) are defined with a gene symbol, whereas the other 7,949 (26.6%) have not yet been formally defined. All probes were engineered using specific lengths (an average of 70 mer) to match the isothermal melting temperatures for superior hybridization performance. Three replicates and three switched replicates were included for each comparison, specifically P8-O/P8-N, P12-O/P12-N, P13-O/P13-N, and P9-N/P30-N. Preliminary statistical analysis was performed with the Significance Analysis of Microarray software (SAM 3.11, Stanford University, Stanford, CA), and the resulting significant genes were subjected to a cutoff with an arbitrary 1.5-fold threshold. The differentially expressed genes identified in specific conditions were compared and annotated using the Database for Annotation, Visualization and Integrated Discovery (DAVID, v6.7), with the whole murine genome as the background, and gene ontology (GO) categorization was performed using a modified Fisher exact test [16]. The p value for each GO category or Kyoto Encyclopedia of Genes and Genomes (KEGG) pathway was calculated as an Expression Analysis Systematic Explorer score [17]. Hierarchical clustering was performed for genes of interest using the Cluster 3.0 program, and the results were transferred onto heat maps using Java Treeview [18]. The complete sets of normalized microarray data were deposited in the NCBI Gene Expression Omnibus, with the accession number GSE38762.

### Reverse transcription and real-time polymerase chain reaction

The expression changes in the genes of interest were confirmed using reverse transcription and quantitative real-time PCR. In brief, total retinal RNA of the OIR models was extracted as described above and reverse transcribed into cDNA using oligo(dT) primers and PrimeScript RT Enzyme (TaKaRa, Dalian, China). Quantitative real-time PCR was performed using the TaqMan method and Probe Real Master Mix (Tiangen Biotech Co., Ltd., Beijing, China) in a 7500 Real-Time PCR System (Applied Biosystems Inc., Foster, CA). Primers and probes (Table 1) were synthesized by GenScript (Nanjing, China), with ribosomal protein L5 (*Rpl5*) as the reference gene. The thermal cycling conditions for all genes were as follows: 95 °C for 10 s followed by 40 cycles at 95 °C for 15 s and 60 °C for 1 min. After analysis with the endorsed software, the fractional cycle number for threshold fluorescence (threshold cycle, Ct) for each reaction was obtained. The average of three duplicates was used to calculate the relative Ct versus *Rpl5* (ΔCt=average Ct_gene_ minus average Ct_Rpl5_) for each sample. The average ΔCt for the three samples in the experimental and control groups was then used to calculate the ΔΔCt of the OIR samples (ΔΔCt=ΔCt_OIR_–ΔCt_control_). The relative expression folds of the OIR samples were calculated as 1/2^ΔΔCt^ [19].

**Table 1:** Primers and probes used for RT-qPCR

Gene symbol (Accession number)	Primer and probe sequence (5′—3′)	Amplicon size (bp)
*Ankrd11*	F: CTGAAGGAGCTGTTCAAGCA	96
NM_001081379	R: TTGCTCACAGGACACAATCA	
	P: TGCTGCAGGCGCAGCTTACC	
*Ankrd37*	F: TGATGTCCAGATTGGCGTAT	120
NM_001039562	R: CTTGCCGTCTGCATACATTT	
	P: CCTGGCACATTCTGGAAAGCCA	
*Anxa2*	F: GTGAAGAGGAAAGGAACCGA	129
NM_007585	R: CTTGATGCTCTCCAGCATGT	
	P: CGCAGTGTGTGCCACCTCCA	
*Mt2*	F: CTGCAAATGCAAACAATGC	116
NM_008630	R: ACTTGTCGGAAGCCTCTTTG	
	P: CCCTGGGAGCACTTCGCACA	
*Tpi1*	F: GAAGAAGTGCCTGGGAGAAC	109
NM_009415	R: CTGTCTGGCAAAGTCGATGT	
	P: AAACCACCTCGGTGCCTGCC	
*Vegfa*	F: CACAGCAGATGTGAATGCAG	111
NM_001025250	R: TTTAAACCGGGATTTCTTGC	
	P: TGACCCTTTCCCTTTCCTCGAACTG	
*Rpl5*	F: GGAAGCACATCATGGGTCAGA	70
NM_016980	R: TACGCATCTTCATCTTCCTCCATT	
	P: TGTGGCAGACTACATGCGCTACC	

## Results

### Comparison of physiologic retinal vasculogenesis and oxygen-induced retinal neovascularization in newborn C57BL/6J mice

**Figure 1:**
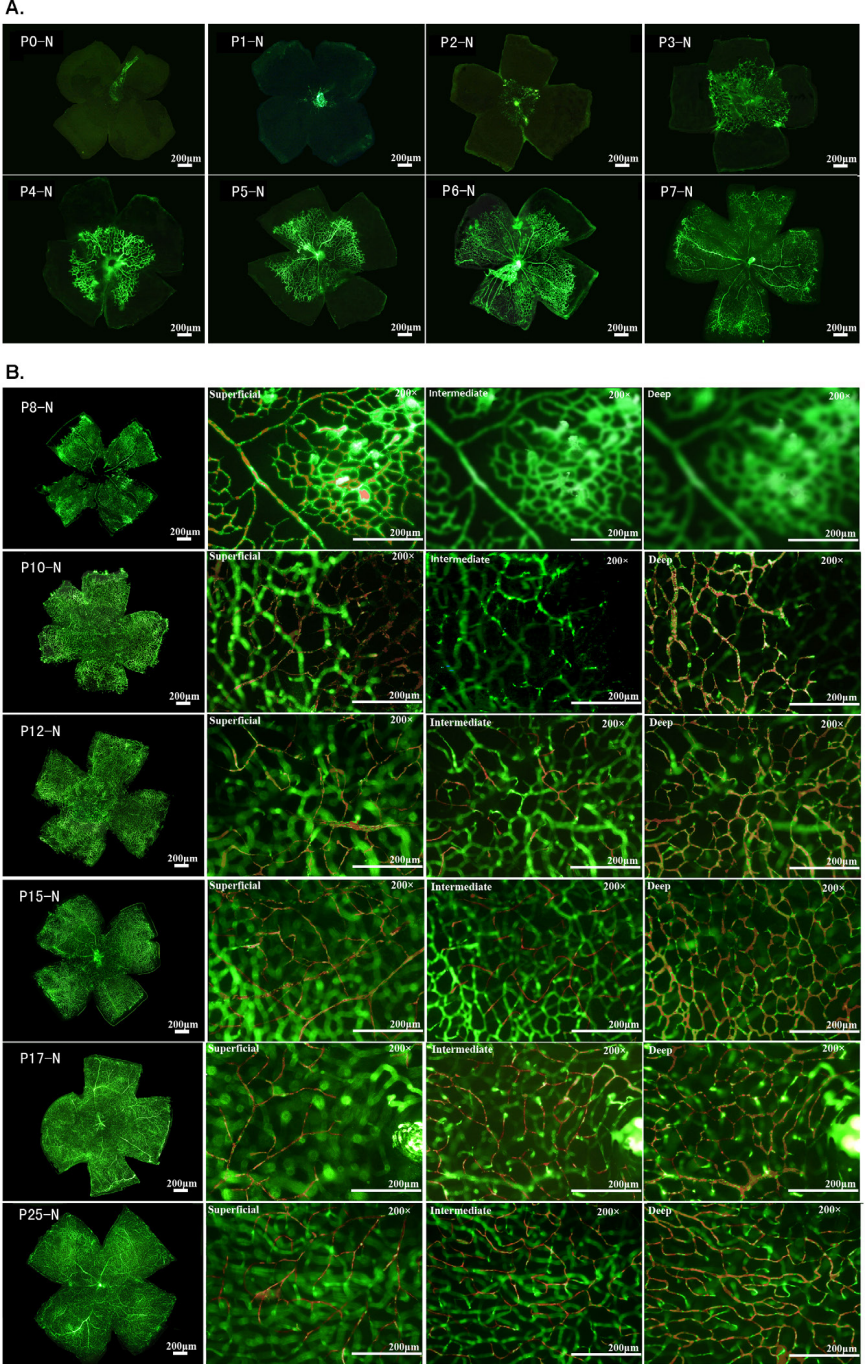
Normal development of the vascular plexuses of C57BL/6J mouse retinas. Following FD-2000S perfusion, retinal whole mounts were examined to assess the distribution of vessels along the width and depth. Great caution was needed to differentiate the vessels at different levels; this differentiation was achieved by continuous slow adjustments of the microscope focus knob. The specified vasculatures (superficial, intermediate, and deep) in the pictures were highlighted by tracing the vessels in red using Adobe Photoshop software. **A**: Observation of retinal vessels as wholemount. From P0 to P7, only superficial retinal vessels were seen to originate from the optic disc, extending rapidly from the inner retina to the peripheral retina during the first week of postnatal development. **B**: Observation of vessels focusing on different layers of the retinal vasculature. From P8, a deep, intermediate vascular plexus started to develop. The deep vascular plexus began forming from vertical vessels diving down from the superficial vascular plexus, expanding from the inner retina into the peripheral retina. In addition, between P8 and P10 the intermediate vascular plexus had not begun to form. At P12, the intermediate vascular plexus was observed, and between P15 and P17, the superficial, intermediate, and deep vascular plexuses were seen all over the retina. After P21, the retinal vessel network was obviously remodeled, especially the intermediate vessel layer, and no obvious changes in the retinal vessels were observed after P25.

We started by monitoring the development of the retinal vasculature in normal C57BL/6J mice from birth (Figure 1). Unlike human beings, mice are completely devoid of blood vessels in the retina at birth (P0-N). Eventually, however, superficial retinal vessels grow radially from the optic disc into the retina, reaching the retinal periphery at P7-N. By P7-N, deep and intermediate vessels are still absent. The deep layer of the retina is formed by P10-N, and the intermediate vessel network is shaped by P12-N. From this point on, the retinal vessels comprise superficial, intermediate, and deep networks over the retina. Certain adjustments or remodeling may occur, as reflected by the slight differences and changes of the vessels, but no more obvious changes were observed in retinal vessels after P25-N.

**Figure 2:**
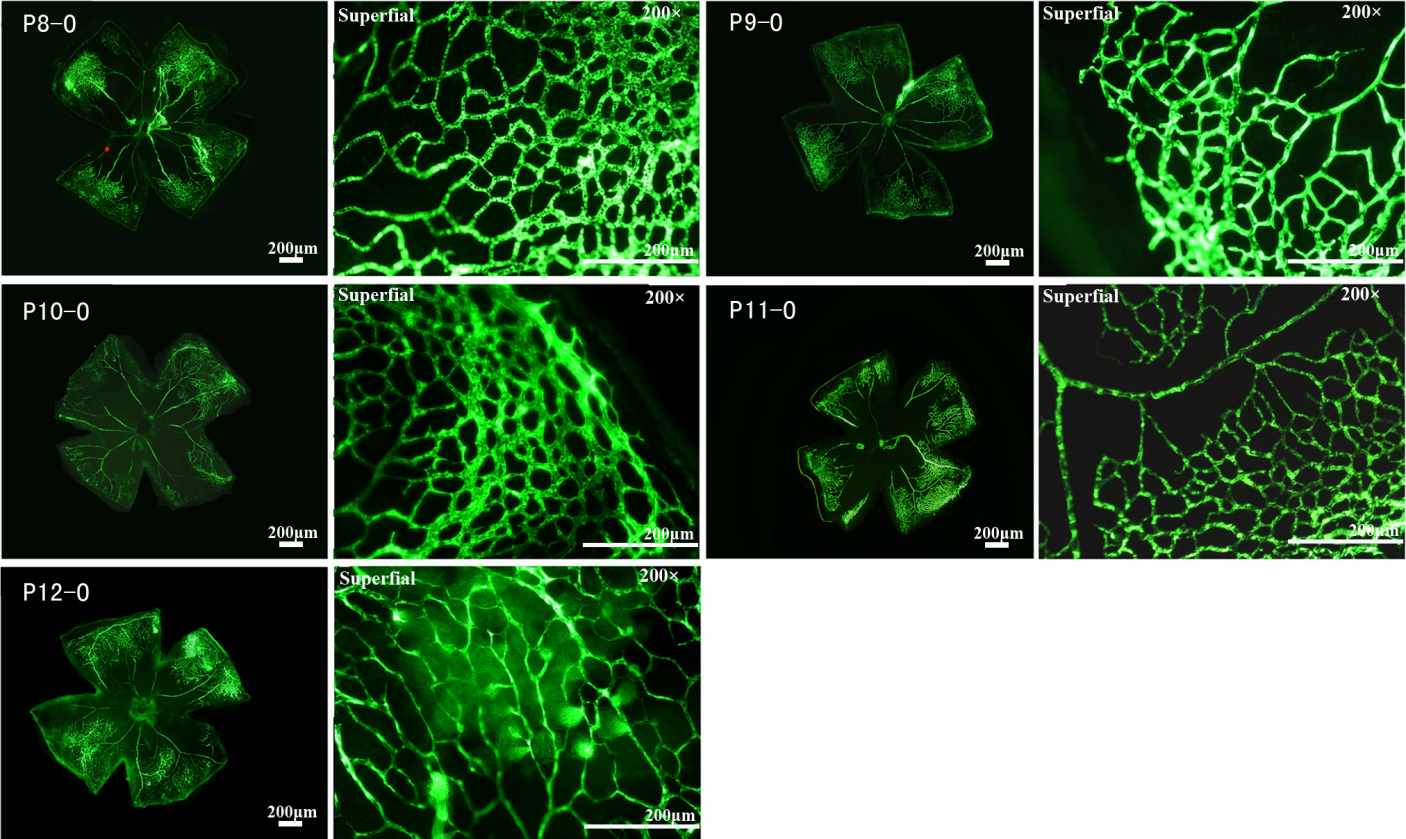
Central non-perfusion area and vasoconstriction under hyperoxic conditions in C57BL/6J mouse retinas. After the mice spent 24 h in a hyperoxic condition, a central area of vasoobliteration (VO) developed rapidly and increased in size until P11, extending further into the peripheral retina, while the size of the central vasoobliteration area decreased slightly from P12. From P8 to P12, only the superficial retinal vascular network was observed, but initiated vascular sprouts were sometimes observed at P12. The development and differentiation of the deep and intermediate vascular plexuses were obviously suppressed. The clear, sharp appearance of all vessels at the single superficial level in all panels also implied the lack of the two other layers of vasculature observed in Figure 1.

**Figure 3:**
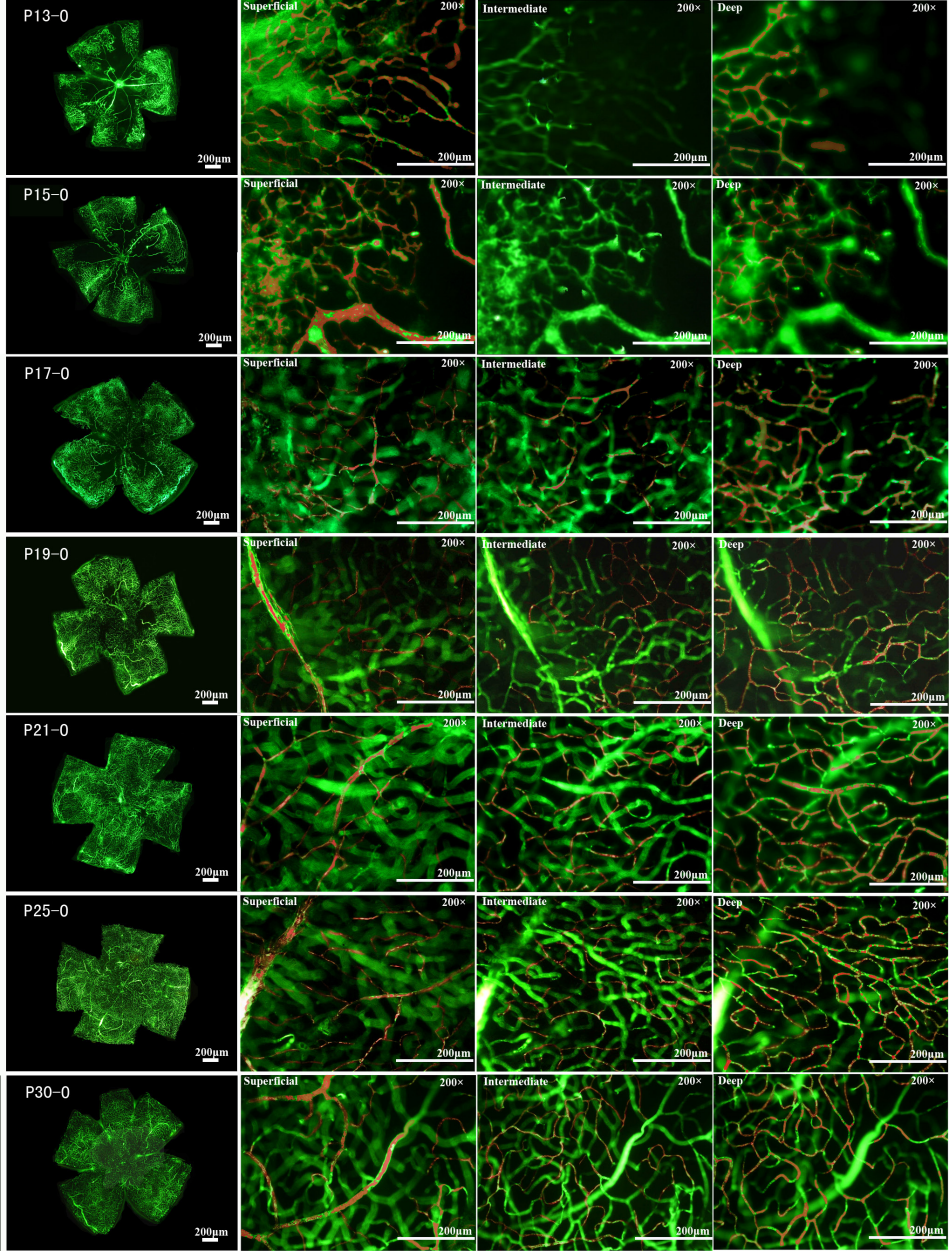
Neovascularization under relative hypoxic conditions in C57BL/6J mouse retinas. After the mice were relocated to room air for 24 h, the size of the vasoobliteration (VO) area decreased slightly, but the deep vascular plexus was observed in the peripheral area, in which area superficial vessels were preserved during hyperoxia. At P15, the size of the VO area continued to decrease, and the deep—but not the intermediate—vascular plexus was observed in the area where superficial vessels were preserved during hyperoxia. The retinal arteries and veins had a dilated and tortuous appearance, and vascular branch sprouts emerged from existing vessels. From P17 to P19, retinal neovascularization progressed rapidly, and numerous vascular sprouts appeared in the transition zone between the vascular and avascular retina. The deep and intermediate plexuses can be observed in almost the same area as the superficial plexus. From P21 to P23, the size of the VO area was small, and neovascularization began to regress. At P25, the VO area was fully revascularized in three layers, neovascularization had completely resolved, and the retinal vessels began to remodel.

The sequential order and pattern of vasculature development are disturbed in the OIR model mice. In general, a large non-perfused area with central vessel constriction was observed 24 h after transfer to the hyperoxic chamber (P8-O). The formation of the non-perfused area may have been caused by the closure and degradation of previous vessels upon hyperoxic challenge. The central retinal vasoobliteration was consistent from P8-O to P12-O, while the peripheral vasoconstriction became aggravated over time. During the entire hyperoxia period, only four to six radial branches of the retinal artery were present in the central area at the superficial level (Figure 2), while intermediate- and deep-layer networks were completely absent over the entire retina (data not shown). While the flat mounts were being prepared, the retinal tissues under hyperoxic conditions were more friable than normal ones, implying that the overall structure of the retina was altered by the hyperoxic treatment. We propose that changes to the retinal vascular system contribute to the overall structural changes of retinas under such conditions. At 24 h after the switch to normoxia, superficial retinal vascularization started along the front line of the perfused area toward the central retina. Aside from the centripetal pattern, the vessels also grew from the superficial layer to the deep layer of the retina. By P15-O, the retina still showed a large area of central vasoobliteration, and the retinal arteries and veins had a dilated and tortuous appearance; furthermore, numerous vascular tufts were present in the transition zone between the non-perfused central retina and the perfused peripheral retina. At P21-O, almost the entire retina was revascularized. From this point on, retinal neovascular tufts regressed gradually and disappeared before P26-O. By P30-O, the OIR retinal vascular network often showed dilated tortuous vessels, abnormal vessel traffic, and uneven vascular distributions (Figure 3), which were absent in the age-matched normal mice. The current study showed that the intermediate networks were absent at P13 and P15, contradicting earlier reports that deep and intermediate vascular networks were observed at P15 [20]. However, no effort was made to pursue this direction, and we have no explanation for these discrepancies.

### Gene expression changes during the early phases of oxygen-induced retinopathy

**Figure 4:**
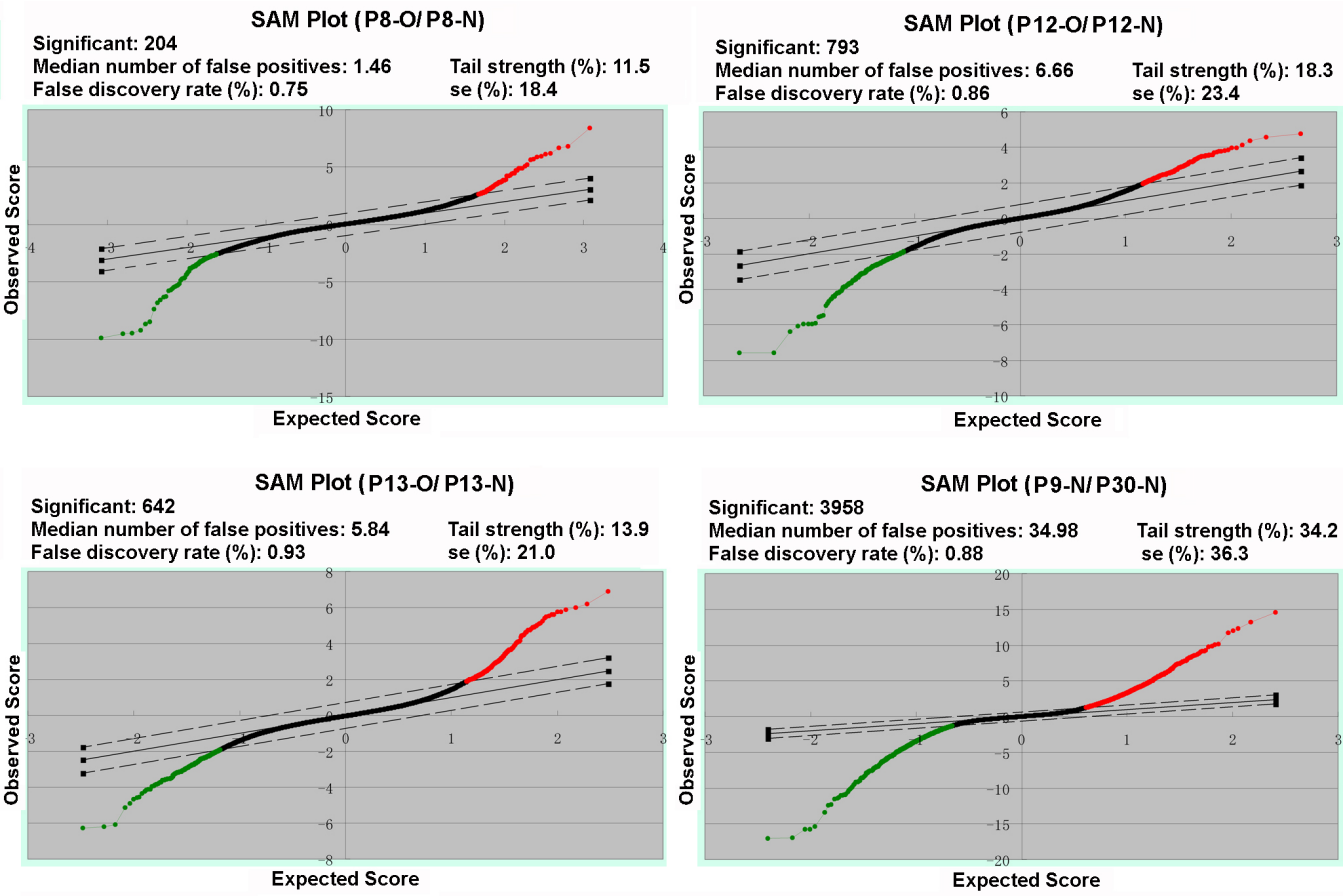
Statistical analysis of microarray (SAM) plots of four comparison pairs. Three arrays were included in each group. The two parallel dashed lines are the cutoff threshold specified by the actual false discovery rate, and the total number of upregulated (red dots) and downregulated (green dots) genes were given for each plot.

**Figure 5:**
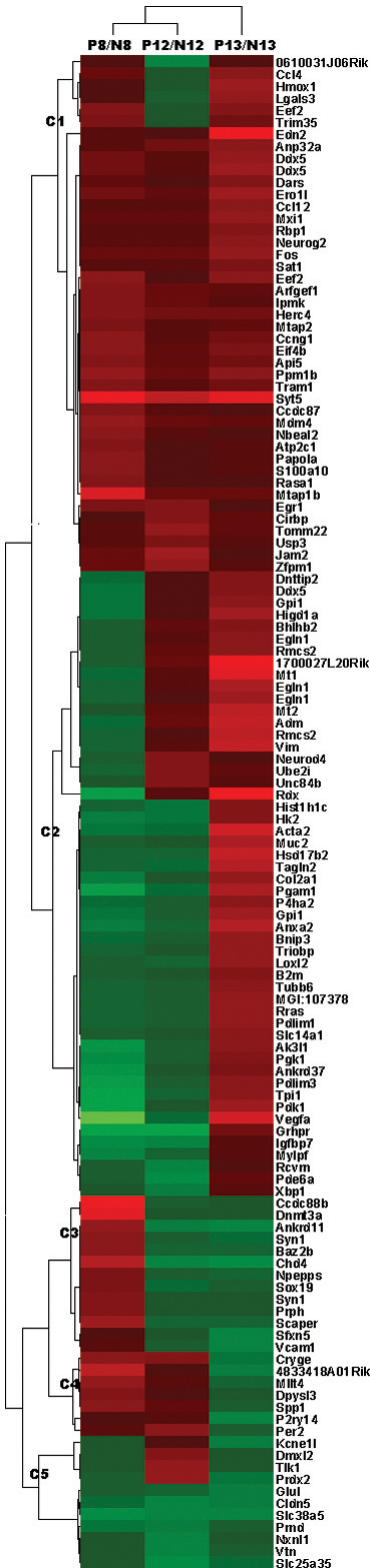
Clustering of the regulated genes that were changed at P8-O/P8-N, P12-O/P12-N, or P13-O/P13-N during the oxygen-induced retinopathy model induction process. Three arrays were included in each group, and the average folds-over-age controls were used. The color bar stands for the log_2_ values of the probe change folds, with red for upregulation and green for downregulation.

To monitor the changes in transcriptomes during the induction and onset of OIR, we compared the gene expression patterns at three critical time points, namely, P8, P12, and P13 (Figure 4). To minimize the potential interference of the expression of genes relating to ocular development in normal mice, the expression patterns of OIR retinas at each time point were compared to those of age-matched animals remaining in normal air from birth, thus making three pairs of samples (P8-O/P8-N, P12-O/P12-N, and P13-O/P13-N). Of an estimated 30,000 probes, 162 changed more than 1.5-fold at one or more time points (Figure 5, Appendix 1). Compared with the genes that showed similar changes at all three time points (like Cluster C1 and C5 in Figure 5), especially at P8-O and P13-O, the genes that manifested contradictory changes at P8-O and P13-O (Clusters C2 and C4 in Figure 5, Table 2) might have done so relative to oxygen pressure changes and may thus provide more information about the development of OIR. When these changed genes were subjected to DAVID analysis, no pathways or GOs were significantly enriched for downregulated genes at any time point or upregulated genes at P12-O. In the 45 upregulated genes with formal nomenclatures for the P8-O/P8-N group, enriched GO was mainly observed at cytoskeleton formation, with *Mtap2*, *Mtap1b*, and *Rasa1* as representative genes. This implies that the cellular response to hyperoxic challenge is mainly related to cytoskeleton remodeling. However, the 62 upregulated genes for P13-O/P13-N have roles in diverse pathological processes (Table 3), indicating that more complex responses are initiated by the hypoxic stimulus. Furthermore, the only enriched KEGG pathway (p value=1.6×10^−4^, enrichment fold=16.9) was glycolysis/gluconeogenesis, which covers glucose phosphate isomerase 1 (*Gpi1*), hexokinase 2 (*Hk2*), phosphoglycerate kinase 1 (*Pgk1*), phosphoglycerate mutase 1 (*Pgam1*), and triosephosphate isomerase 1 (*Tpi1*). Unlike *Vegfa* (Table 2 and Table 3), however, another best-known hypoxic responsive gene, *Hif1α*, did not show significant changes in our study. The P8-O/P8-N and P13-O/P13-N ratios for *Hif1α* were 1.0491±0.1454 and 1.0493±0.0977, respectively.

**Table 2:** Genes showing opposite changes at P8 and P13 as compared with age controls.

Gene symbol	P8-O/P8-N ratio	P13-O/P13-N ratio
*Chd4*	2.1374	0.5983
*Ankrd11*	1.7291	0.6465
*Anxa2*	0.6669	2.1625
*Pgk1*	0.6066	1.5450
*Ankrd37*	0.5776	1.5206
*Ak3l1*	0.5606	1.7040
*Pgam1*	0.5438	2.0699
*Rdx*	0.5414	10.3585
*Pdlim3*	0.5330	1.6370
*Pdk1*	0.5242	1.8361
*Tpi1*	0.5030	1.6187
*Vegfa*	0.2877	2.4823

**Table 3:** Enriched gene ontology terms in upregulated genes in P8-O/P8-N and P13-O/P13-N.

GO Term	P Value	Genes	Fold Enrichment
P8-O/P8-N			
regulation of cytoskeleton organization	0.012034	*Mtap2, Mtap1b, Rasa1*	17.157
P13-O/P13-N			
glycolysis	1.41E-05	*Tpi1, Pgam1, Hk2, Pgk1, Gpi1*	32.853
glucose metabolic process	1.05E-04	*Pdk1, Tpi1, Pgam1, Hk2, Pgk1, Gpi1*	12.390
generation of precursor metabolites and energy	0.001813	*Tpi1, Pgam1, Hk2, Ero1l, Pgk1, Gpi1*	6.646
circulatory system process	0.00624	*Acta2, Edn2, Hmox1, Vegfa*	10.418
nitric oxide mediated signal transduction	0.006759	*Mt2, Mt1*	289.106
angiogenesis	0.01024	*Hmox1, Vegfa, Gpi1, Anxa2*	8.695
vascular process in circulatory system	0.012388	*Acta2, Edn2, Vegfa*	17.347
response to oxygen levels	0.020371	*Hmox1, Vegfa, Bnip3*	13.344
immune response	0.020881	*Ccl12, Rmcs2, Vegfa, Bnip3, Ccl4, B2m*	3.683
vasoconstriction	0.043145	*Acta2, Edn2*	44.478
negative regulation of multicellular organismal process	0.045008	*Hmox1, Vegfa, Anxa2*	8.673
extracellular matrix organization	0.045821	*Lgals3, Col2a1, Anxa2*	8.587

### Genes involved in the physiologic development of normal retinal vasculature

Due to the obvious difference between normal retinal vasculature and oxygen-induced RNV, we also monitored the genes that might be involved in normal retinal development by comparing the gene patterns at P9-N against those of P30-N. Among all probes corresponding to defined transcripts with formal nomenclature, 135 were upregulated and 231 were downregulated 1.5-fold in P9-N/P30-N (Appendix 2). Among the upregulated genes, the gap junction and Fc gamma R-mediated phagocytosis were the two main enriched KEGG pathways, while for the downregulated genes, the main pathways were starch and sucrose metabolism and purine metabolism (Table 4). Taking P13-O/P13-N into account, among all 419 defined probes that changed in at least one condition (P13-O/P13-N, P9-N/P30-N), nine showed consistent changes, and eight showed opposite changes, while the other 402 changed in only one condition (Table 5). The 17 genes that showed opposite or concert changes are listed in Table 6, and the representatives of the 53 probes that changed only at P13-O/P13-N but not at P9-N/P30-N and their corresponding genes are listed in Table 7 (and Appendix 3 for all probes). Again, *Vegfa*, the most extensively studied proangiogenic factor, was upregulated in the P13-O/P13-N group but remained unchanged in the P9-N/P30-N group, indicating that this gene might be more relevant with pathological neovascularization but not with physiologic angiogenesis. The changes in several genes of interest were further confirmed with real-time PCR, and good agreement between the microarray and PCR measurements was observed (Table 8).

**Table 4:** Enriched pathways in P9-N/P30-N.

Term	P Value	Genes	Fold Enrichment
**Downregulated pathways**			
Starch and sucrose metabolism	0.008	*Pygm, Gaa, Hk2, Hk1*	9.516
Purine metabolism	0.034	*Pde6a, Pde6b, Adssl1, Pde8a, Pde6h, Pde6g*	3.273
**Upregulated pathways**			
Gap junction	0.028	*Tubb2b, Tubb2a, Tubb5, Tuba1a*	5.931
Fc gamma R-mediated phagocytosis	0.039	*Marcksl1, Marcks, Arpc5, Pik3r1*	5.205

**Table 5:** Matching of genes that changed in physiologic (P9-N/P30-N) or pathological (P13-O/P13-N) angiogenesis.

	**P9-N/P30-N**		
**P13-O/P13-N**	Up-regulated	Down-regulated	Unchanged
Up-regulated	6	5	51
Down-regulated	3	3	2
Unchanged	126	223	21554

**Table 6:** Genes that manifested concert or opposite changes at P9-N/P30-N and P13-O/P13-N.

Gene symbol	Definition	P13-O/P13-N ratio	P9-N/P30-N ratio
*Kcne1*	potassium voltage-gated channel, Isk-related subfamily, member 1	0.6592	2.3086
*Vcam1*	vascular cell adhesion molecule 1	0.6341	1.9022
*Slc38a5*	solute carrier family 38, member 5	0.6191	1.7993
*Rdx*	radixin	10.3585	1.6608
*Tagln2*	transgelin 2	2.1198	1.6158
*Col2a1*	collagen, type II, alpha 1	1.7254	1.9554
*Neurog2*	neurogenin 2	1.7086	1.6937
*Rbp1*	retinol binding protein 1, cellular	1.6143	1.7148
*Sat1*	spermidine/spermine N1-acetyl transferase 1	1.5006	2.1131
*Glul*	glutamate-ammonia ligase 1	0.6594	0.2625
*P2ry14*	purinergic receptor P2Y, G-protein coupled, 14	0.6286	0.6476
*Sfxn5*	sideroflexin 5	0.6238	0.5462
*Mt1*	metallothionein 1	2.6682	0.3433
*Mt2*	metallothionein 2	2.2200	0.6344
*Fos*	FBJ osteosarcoma oncogene	1.7431	0.4556
*Bhlhb2*	Class B basic helix–loop–helix protein 2	1.5353	0.4539
*Hk2*	hexokinase 2	1.5251	0.1495

**Table 7:** Genes that changed at P13-O/P13-N only but remained stable at P9-N/P30-N.

Gene symbol	Gene name	P13-O/P13-N ratio	P9-N/P30-N ratio
*Adm*	adrenomedullin	2.2952	1.2184
*Anxa2*	annexin A2	2.1625	1.2005
*Ccl12*	chemokine (C-C motif) ligand 12	1.6336	1.0920
*Ccl4*	chemokine (C-C motif) ligand 4	1.6343	1.0484
*Chd4*	chromodomain helicase DNA binding protein 4	0.5983	1.4473
Ddx5*	DEAD (Asp-Glu-Ala-Asp) box polypeptide 5	1.8907, 1.7185, 1.5464	1.1269, 1.0889, 0.8610
*Edn2*	endothelin 2	2.8626	0.8801
Egln1*	EGL nine homolog 1 (C. elegans)	1.8848, 2.0157, 1.6188	0.9181, 0.9040, 0.9575
Ero1l*	ERO1-like (S. cerevisiae)	1.8372, 1.9127	0.8408, 0.7613
*Gpi1*	glucose phosphate isomerase 1	1.6661	0.8129
*Hmox1*	heme oxygenase (decycling) 1	1.8236	1.1132
*Muc2*	mucin 2	2.0306	0.9220
*Pgam1*	phosphoglycerate mutase 1	2.0699	0.8084
*Syt5*	synaptotagmin V	2.7145	0.7828
*Tpi1*	triosephosphate isomerase 1	1.6187	0.6997
*Vegfa*	vascular endothelial growth factor A	2.4823	1.2186
*Vim*	vimentin	2.3312	1.4946

Note *: These genes have two or three probes in this array setting, thus have more than one ratio in each condition.

**Table 8:** Confirmation of changes of six genes by RT-qPCR

Gene symbol	P8-O/P8-N PCR ratio	P8-O/P8-N Chip ratio	P13-O/P13-N PCR ratio	P13-O/P13-N Chip ratio	P9-N/P30-N PCR ratio	P9-N/P30-N Chip ratio
*Ankrd11*	2.2830	1.7291	1.5625	0.6465	0.5672	1.2766
*Ankrd37*	0.3149	0.5776	1.8058	1.5206	0.5643	1.0405
*Anxa2*	0.4648	0.6669	3.5604	2.1625	3.2840	1.2005
*Mt2*	5.3914	1.0532	2.1010	2.2200	0.4132	0.6344
*Tpi1*	0.5288	0.5030	2.6888	1.6187	0.7075	0.6997
*Vegfa*	0.7057	0.2877	4.8914	2.4823	0.9922	1.2186

## Discussion

Three previous studies [10-12] employed microarray technology to study gene expressions in OIR models. Although similar OIR models were used, huge differences exist among the previous reports and our own. Ishikawa [11] noted that eight genes were upregulated and 75 downregulated by greater than 1.5-fold in hyperoxic retinas over normoxic retinas (P12-O/P12-N), while we recorded 22 upregulated and 15 downregulated probes in the same setting. These two sets of microarray data share only four downregulated genes (*Cldn5*, *Igfbp7*, *Slc38a5*, and *Vtn*) and no upregulated genes for P12-O/P12-N. Unable to address all such discrepancies among all studies, this discussion focused on limited examples, such as *Hif1*.

*Hif1* is a factor most often proposed as responsible for hypoxic stress, including in angiogenesis. To form bioactive *Hif1*, *Hif1α* must dimerize with *Hif1β*, and modulation of *Hif1* activity is mainly achieved with modification (e.g., hydrocxylation or ubiquination) of *Hif1α* subunits and the dimerization process of *Hif1α* with *Hif1β*, though alterations of *Hif1α* gene transcriptions also contribute to modulating overall *Hif1α* activity in certain conditions. Both mechanisms are responsive to oxygen tension-related stimuli (refer to [21,22] for an extensive review). In the OIR models discussed here, *Hif1α* was upregulated by 47,162-fold at P13 in Sato’s study [10] and 2.22-fold at P12.5 (e.g., 12 h after returning to normoxic conditions) in Ishikawa’s study [11], but showed no significant changes in either our P13-O/P13-N array or in Recchia’s study [12]. Not knowing the exact cause of such a discrepancy with any gene in these parallel independent studies, we believe that several variations among the experiments might contribute. First, Sato and Ishikawa used C57BL/6N mice, Recchia used C57BJ/6L mice, and we used C57BL/6J mice. Although OIR strain dependence is already recognized [23], the genetic differences among various sub-strains had also been partially defined [24,25]. Second, all four studies used different array platforms, and significant inconsistencies among different microarray platforms are sometimes expected [24,26]. Moreover, the variations in the in-house experiment protocols in different laboratories (e.g., the time used for animal bedding changes, the time from mice euthanasia to RNA extraction, the pooling of samples from animals, etc.) may all contribute to the different results from these studies.

Similarly, the time courses of oxygen tension changes, as well as the detection time points, need to be considered when interpreting the results with strictly controlled genes like *Hif1*. For example, in a study using epidermal keratinocytes, Weir et al. [27] reported that prolonged (chronic) hypoxia (1% O_2_ for 18 h) leads to the downregulation of *Hif1α* but the upregulation of classical *Hif1* targets such as *Vegfa*. This may, in part, explain the *Vegf* upregulation (Table 2 and Table 8) in the absence of *Hif1* changes in the current study. Similarly, several other genes, such as *Ctgf*, *bFgf*, *Pdgf*, and *Ang1*, are also known to be involved in modulating *Vegf* expression in other situations; however, in the current study, none showed significant changes in the hyperoxia (P8-O/P8-N group) or hypoxia (P13-O/P13-N) challenges (data not shown). Collectively, the roles of *Hif1α*, *Vegf*, or other genes in OIR deserve more extensive investigation.

Because few changes in classic pro- or antiangiogenic factors were observed in our study, clustering of the regulated genes in OIR (P13-O/P13-N) did not reveal any angiogenic pathways. However, the enrichment of some angiogenesis- or glycolysis-related gene ontology terms in the clustering assay (Table 3) was suggestive. For example, *Hmox1* was grouped with ontology terms of the circulatory system process, angiogenesis, response to oxygen levels, and the negative regulation of the multicellular organismal process (Table 3); its upregulation in P13-O/P13-N thus supports a potential role in OIR. Pursuing these genes or gene ontology terms may lead to a comprehensive understanding of neovascularization in the OIR context. Similarly, the enrichment of glucose metabolism-related genes in P13-O/P13-N (Table 3) suggested that the interactions among hypoxia, glucose, and *Hif1* also exist to some extent in the OIR model, as in the case of tumors [28,29]. Although the ultimate results of *Hif1* action may partially depend on glycolysis [30], glucose levels in return affect the responses of *Hif1* expression to hypoxia [31,32]. Again, the absence of changes in *Hif1a* expression and significant changes to glycolysis-related genes (Table 3) in our study reflect the complexity of the interactions between *Hif1* and glycolysis in this experimental condition.

Another issue that deserves discussion is the differential behaviors of genes in the P9-N/P30-N and P13-O/P13-N settings, which were assumed to represent physiologic and pathological vessel formation, respectively. The genes that relate to angiogenesis but show differential changes in P13-O/P13-N and P9-N/P30-N (Table 6 and Table 7) might imply that hypoxia-induced RNV and physiologic angiogenesis are under the control of different regulating networks. This hypothesis is in line with the findings in a recent study, where Chabot showed that some molecules (e.g., CD160) were selectively expressed in newly formed blood vessels in tumors but not in healthy tissues, and that antibodies against these molecules reduced neovascularization in several animal models, including the murine OIR model [33]. Similarly, we propose that the genes upregulated in the P13-O/P13-N setting, regardless of changes in the P9-N/P30-N setting, may pose potential targets for managing neovascularization. Examples of such genes include *Hk2*, metallothionein 1 (*Mt1*), and *Mt2* (Table 6). *Hk2* has long been known as a glycolytic enzyme, and has recently been proven to be involved in tumor or metabolic disorders [34-36] or hypoxia-induced angiogenesis in these conditions [37]. *Mt* is a family of cysteine-rich, low molecular weight proteins, the gene expression of which is induced by various stimuli, such as metal exposure, oxidative stress, and cytokines. *Mts* are thus involved in various injury- or stress-related pathological processes such as ischemia [38], ischemia-reperfusion injury [39], wound healing [40], and inflammation or immune responses [41,42]. Recent studies using *Mt* knockout mice have shown that *Mt1* and *Mt2* are involved in angiogenesis by functioning in endothelial cells, smooth muscle cells, and macrophages [43,44]. Our observation that *Hk2*, *Mt1*, and *Mt2* were upregulated 24 h after switching to relative hypoxic conditions provided further evidence for the roles of *Mt* in pathological angiogenesis; hence, *Mt* is potentially useful in managing neovascularization-related disease. For similar reasons, several other genes, including annexin A2 and endothelin 2 (Table 7), are being studied in this laboratory for their potential usefulness in managing RNV (data not shown).

In summary, the data described here bring insights into hypoxia-induced retinopathy, and contribute to our understanding of the clinical situation of OIR. By monitoring the development of retinal vasculature in a dynamic pattern under physiologic and OIR conditions, we showed that hyperoxic tension not only led to the degeneration of superficial retinal vessels but also delayed the development of the deep and intermediate layers of vasculature (Figure 2 and Figure 3). However, thus far, few studies have been conducted on the contributions of individual layers to the overall physiologic function of the retina, as well as abnormalities of which layers do the most damage to retinal organization and function. Microarray assays of retinas revealed the complexity of molecular networks underlying the development of OIR. Investigating dynamic changes in specific genes such as *Hk2*, *Mt1*, or *Mts2* might help to answer these questions, and more new stage- or site-specific protocols or therapeutics can thus be developed to intervene in neovascularization development, providing novel therapies for related diseases.

## Appendix 1.

Summary of the differentially expressed genes in the three comparison groups. To access the data, click or select the words “Appendix 1.”

## Appendix 2.

Summary of the differentially expressed genes in the P9-N/P30-N comparison. To access the data, click or select the words “Appendix 2.”

## Appendix 3.

All probes that changed only at P13-O/P13-N but remained stable at P9-N/P30-N. To access the data, click or select the words “Appendix 3.”
